# Selective carbon dioxide sorption and heterogeneous catalysis by a new 3D Zn-MOF with nitrogen-rich 1D channels

**DOI:** 10.1038/s41598-017-17584-8

**Published:** 2017-12-07

**Authors:** Hyun-Chul Kim, Seong Huh, Sung-Jin Kim, Youngmee Kim

**Affiliations:** 1 Department of Chemistry and Protein Research Center for Bio-Industry, Hankuk University of Foreign Studies, Yongin, 17035 Korea; 2 Department of Chemistry and Nano Science, Ewha Womans University, Seoul, 03760 Korea

## Abstract

We prepared a new *C*_2h_-symmetric bridging ligand, 3,3′-(pyrazine-2,5-diyl)dibenzoic acid (3,3′-PDBA), through a Suzuki coupling reaction. 3,3′-PDBA contains a central pyrazine ring instead of the phenyl ring of 3,3′-terphenyldicarboxylic acid (3,3′-TPDC). Despite the geometrical similarity of the two bridging ligands, the reaction between Zn(NO_3_)_2_·6H_2_O and 3,3′-PDBA in the presence of 1,4-diazabicyclo[2.2.2]octane (DABCO) yielded a structurally different Zn-based metal-organic framework (Zn-MOF). The Zn-MOF, [Zn_2_(3,3′-PDBA)_2_(DABCO)_1.5_]·2DMF·H_2_O, had two-dimensional (2D) layers, and the interlocked 2D layers formed a porous 3D framework. Interestingly, one of the two available N atoms of DABCO remained intact. The uncoordinated N atoms of the dangling DABCO ligand and the pyrazinyl N atoms of the 3,3′-PDBA bridging ligand were fully exposed toward the 1D channels. Therefore, the 1D channels represented a highly nitrogen-rich environment. Gas sorption analysis indicated that the Zn-MOF was selective for adsorption of CO_2_ at 196 K over N_2_ (77 K) and H_2_ (77 K). The exceptionally high zero surface coverage heat of CO_2_ adsorption (*Q*_st_ = 79.5 kJ mol^−1^) was attributable to the openly accessible multiple Lewis basic sites in the nitrogen-rich 1D channels. Zn-MOF also showed good Lewis base catalytic activities in three model aldol-type reactions with good recyclability due to chemically accessible 3° amine sites.

## Introduction

The incorporation of chemically accessible Lewis basic sites into the channels of metal-organic frameworks (MOFs) is still challenging but significantly important to develop new functional crystalline porous materials for diverse applications, such as selective CO_2_ capture and heterogeneous catalysis^1–6^. Additionally, Lewis basic sites inside channels can be further functionalized on demand for various applications^7–10^. We recently reviewed several novel approaches for the generation of accessible Lewis basic sites inside MOF channels^11^. Most methods for the generation of intact Lewis basic sites rely on the use of multitopic bridging ligands containing extra Lewis basic sites that cannot easily be involved in coordination to metal ions during MOF synthesis. The use of pyridine-3,5-dicarboxylate (3,5-PDC) and 2,2′-bipyridine-5,5′-dicarboxylate (bpydc) ligands are good examples to provide free pyridyl and 2,2′-bipyridyl moieties^11^.

Despite these encouraging examples, the generation of intact 3° amine-based Lewis basic sites is rather challenging due to their enhanced basicity. Generally, there are two available methods to generate such 3° amine functionality in MOF channels. First is the generation of open metal sites in pre-assembled MOFs followed by the coordination of bifunctional 3° amine compounds, such as 1,4-diazabicyclo[2,2,2]octane (DABCO) or *N*,*N*-diethylenediamine^2,5,12^. Only one of the two available N donor atoms of diamines tends to coordinate to the open metal site, and the other N atom remains uncoordinated, producing 3° amine-functionalized open channels. In theory, this method is straightforward; however, the process inherently involves multiple steps, and the complete introduction of diamines into all open metal sites is practically impossible.

The second method is the special design of bridging ligands, which can induce constrained framework structures in which bifunctional 3° diamines are only able to coordinate through one of the two available N atoms. In this approach, diamines can be directly added together into the reaction mixture for MOF synthesis. We successfully utilized this approach to prepare the first example of structurally characterized DABCO-functionalized Zn(3,3′-TPDC)-MOF (Zn-MOF **1**), [Zn(3,3′-TPDC)(DABCO)]·DMF·2H_2_O where 3,3′-TPDC is the *C*_2h_-symmetric terphenyl-3,3′-dicarboxylate linker^13^. Each layer of two-dimensional (2D) Zn-MOF **1** noncovalently packs to form a 3D-like framework with permanent microporosity. In the structure of Zn-MOF **1**, orderly aligned DABCO ligands are located in the 1D cage-like channels. Notably, the intact N atoms of the dangling DABCO ligands orient toward the channels. As a result, solvent-free Zn-MOF **1** with openly accessible 3° amine sites is both a good CO_2_ sorbent with high selectivity over N_2_ and H_2_ and a substrate-selective heterogeneous catalytic system for aldol-type C–C bond forming reactions with good recyclability^13,14^.

**Figure 1:**
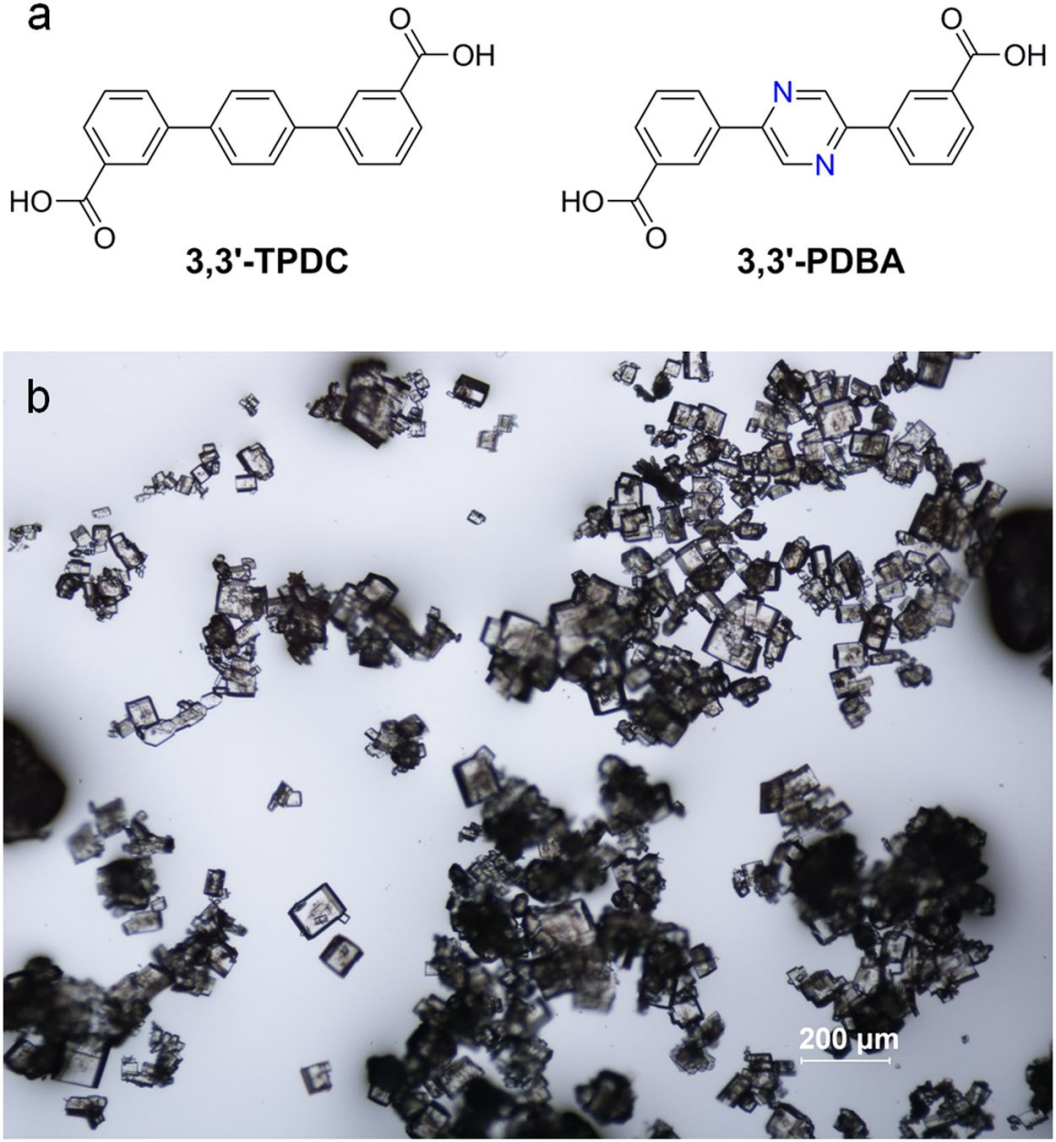
Chemical structures of the ligands and photographic image of the MOF crystals. (**a**) Chemical structures of 3,3′-TPDC and 3,3′-PDBA. The nitrogen atoms in 3,3′-PDBA are highlighted in blue. (**b**) Optical microscopic image of crystals of as-prepared Zn-MOF **2**.

Stimulated from these interesting results, we attempted to modify the structure of the 3,3′-TPDC linker in Zn-MOF **1** to tune the cage-like 1D channels to contain more Lewis basic sites. After careful examination of the channel structure of Zn-MOF **1** (Fig. S1), it can be easily seen, once the central phenyl ring of the 3,3′-TPDC linker is replaced by a pyrazinyl moiety using a 3,3′-PDBA (3,3′-(pyrazine-2,5-diyl)dibenzoic acid) linker, the 1D channels become more nitrogen-rich. To achieve this goal, herein we prepare a new *C*_2h_-symmetric ditopic bridging 3,3′-PDBA ligand, as shown in Fig. 1a. The geometrical structure of 3,3′-PDBA is the same as 3,3′-TPDC. However, 3,3′-PDBA contains a nitrogen-rich pyrazinyl ring instead of the central benzene ring of 3,3′-TPDC. A new Zn(3,3′-PDBA)-MOF, [Zn_2_(3,3′-PDBA)_2_(DABCO)_1.5_]·2DMF·H_2_O (Zn-MOF **2**), containing 3,3′-PDBA and DABCO was prepared and structurally characterized by single-crystal X-ray analysis. The crystal structure revealed that Zn-MOF **2** contains nitrogen-rich channels due to the presence of openly accessible 3° amine and pyrazinyl sites. These unique nitrogen-rich channels can enhance the Lewis acid-base interaction between CO_2_ and basic sites. The gas sorption, adsorptive iodine and CS_2_ encapsulation properties and heterogeneous catalytic activities of newly prepared Zn-MOF **2** are investigated.

## Results and Discussion

The new *C*_2h_-symmetric dicarboxylate-based bridging ligand, 3,3′-(pyrazine-2,5-diyl)dibenzoic acid (3,3′-PDBA), was prepared through the Suzuki coupling reaction between 2,5-dibromopyrazine and 3-(methoxycarbonyl)phenylboronic acid followed by acid hydrolysis of the resulting carboxy-protected ester compounds, as shown in Fig. S2. The resulting ester compounds are a mixture of esters containing methoxy and ethoxy protecting groups. The ethoxy-protected ester compound may be generated during the Pd-catalyzed Suzuki coupling reaction through the transesterification of the methoxy-protected ester compound in ethanol. The new Zn-MOF, [Zn_2_(3,3′-PDBA)_2_(DABCO)_1.5_]·2DMF·H_2_O (Zn-MOF **2**), was prepared from the thermal reaction of Zn(NO_3_)_2_·6H_2_O with 3,3′-PDBA and DABCO in DMF. Transparent well-shaped block crystals suitable for single-crystal X-ray crystallography were directly produced, as shown in Fig. 1b.

**Figure 2:**
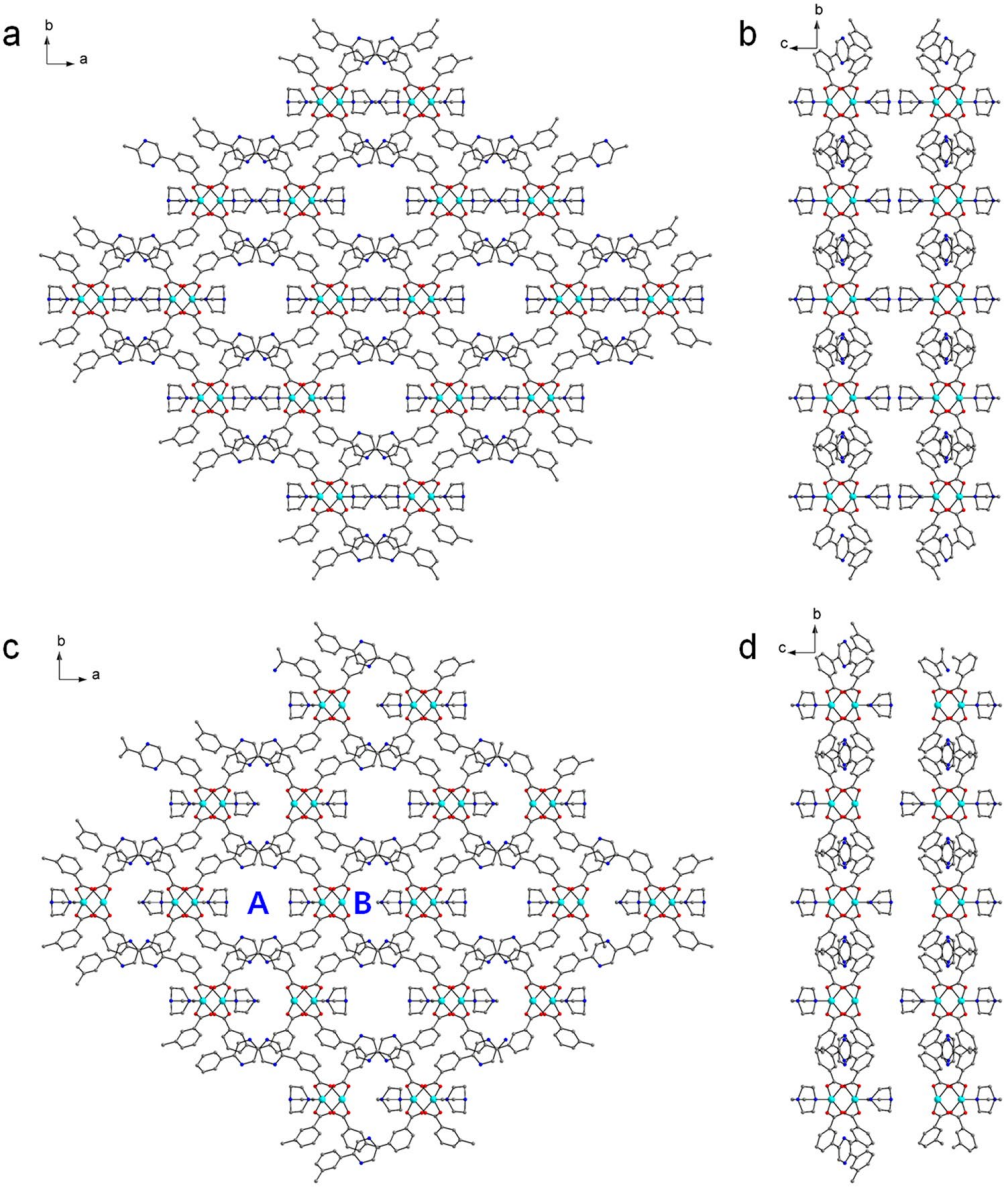
Crystal structures. (**a**) 2D structure of Zn-MOF **2** containing Zn_2_(CO_2_)_4_ dinuclear SBUs with axial DABCO ligands shown along the *c*-axis. (**b**) The same structure viewed along the *a*-axis. (**c**) 2D sheet containing two Zn^II^ ions of paddle-wheel dinuclear SBUs coordinated by DABCO ligands alternatively shown along the *c*-axis. Large pore ‘A’ and small pore ‘B’ are indicated. (**d**) The same structure viewed along the *a*-axis.

**Figure 3:**
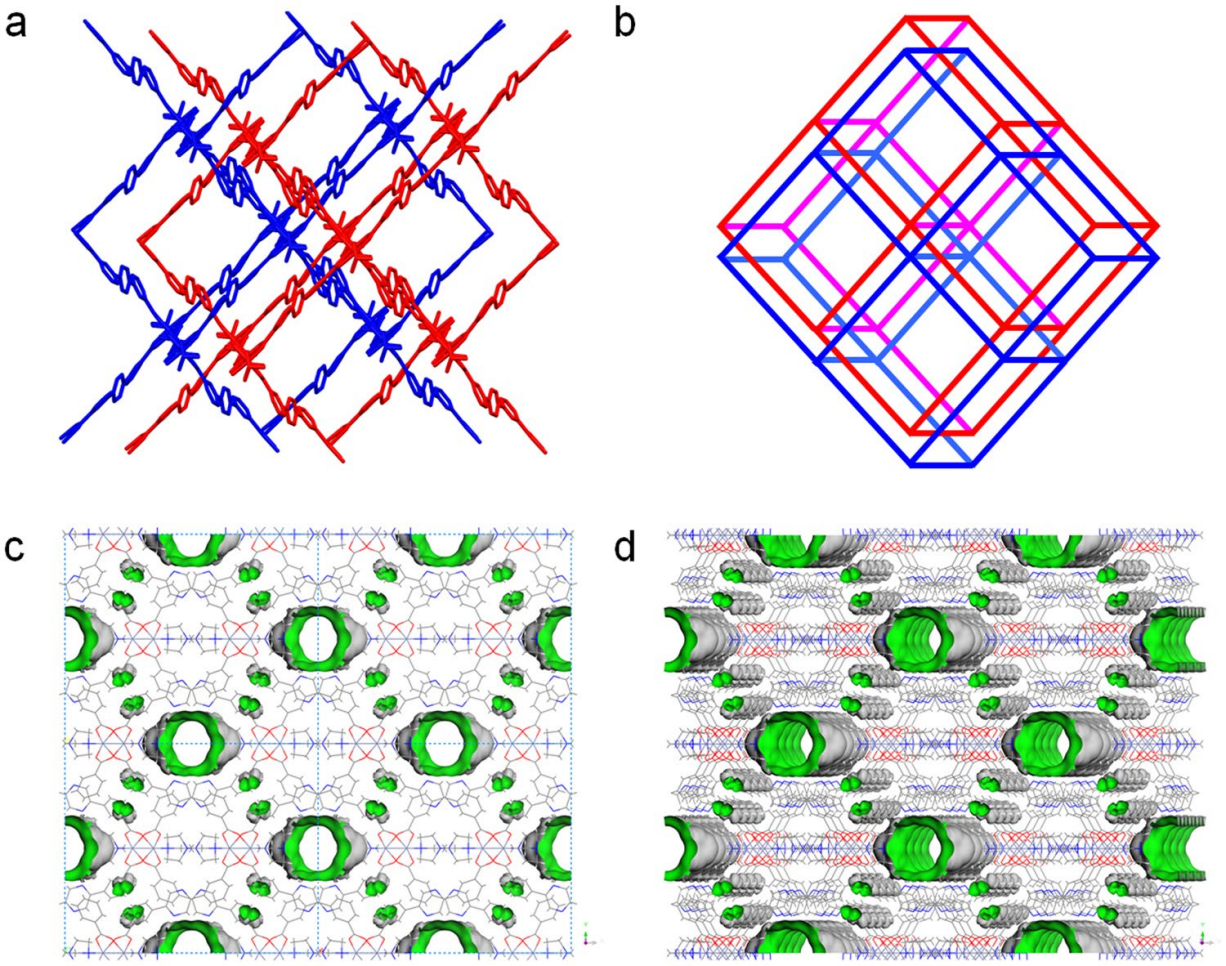
Framework structures and Connolly surfaces. (**a**) Interlocked 2D sheets of solvent-free Zn-MOF **2**. Only two 2D sheets are shown for clarity. Each 2D sheet is interlocked with two other 2D sheets to form an infinite 3D framework. (**b**) Schematic illustration of the interlocked structure shown in (**a**). (**c**) Connolly surface of solvent-free Zn-MOF **2** along the *c*-axis. (**d**) The tilted view of the Connolly surface. The surface was probed by a probe radius of 1.4 Å (Materials Studio 4.4).

Zn-MOF **2** crystallized in the monoclinic *C2/m* space group. Carboxylates of the 3,3′-PDBA linker bridge paddle-wheel dinuclear secondary building units (SBUs) to form 2D sheets (Fig. 2). These Zn_2_(CO_2_)_4_ SBUs are also axially coordinated by DABCO ligands. Notably, Fig. 2a shows that the two middle DABCO ligands are shown connected through N-N bonds, which were derived from the symmetry of the *C2/m* space group. One of the Zn^II^ ions of the dinuclear SBU is coordinated by a DABCO ligand, and the other Zn^II^ ion is also coordinated by half of a DABCO ligand. We can draw the 2D sheet containing two kinds of dinuclear SBUs alternatively: one unit is axially coordinated by DABCO ligands on both sides, and the other is axially coordinated by DABCO on one side, as shown in Fig. 2b. As a result, there are two different types of 1D micropores, large pore ‘A’ and small pore ‘B’, as depicted in Fig. 2c. The Connolly surface shown in Fig. 3 only indicates the large 1D micropore among the two available micropores. Very well-defined 1D channels are identified. The coordination geometry of the Zn^II^ ions is square pyramidal constructed by four carboxylate O atoms and one or half of a DABCO N atom. The PLATON analysis indicated that the solvent-accessible void volume of the solvent-free Zn-MOF **2** was 11.8% (549.0 Å^3^/ 4635.8 Å^3^). Despite the relatively low void space of the solvent-free Zn-MOF **2**, it was clearly revealed that the uncoordinated N atoms of the dangling DABCO ligands are orderly aligned along the 1D channels and that they are well exposed to the channels, as depicted in Fig. 3. Moreover, the N atoms of the pyrazine moieties in 3,3′-PDBA are also well exposed to the channels. Thus, Lewis basic nitrogen-rich 1D channels are realized.

**Figure 4:**
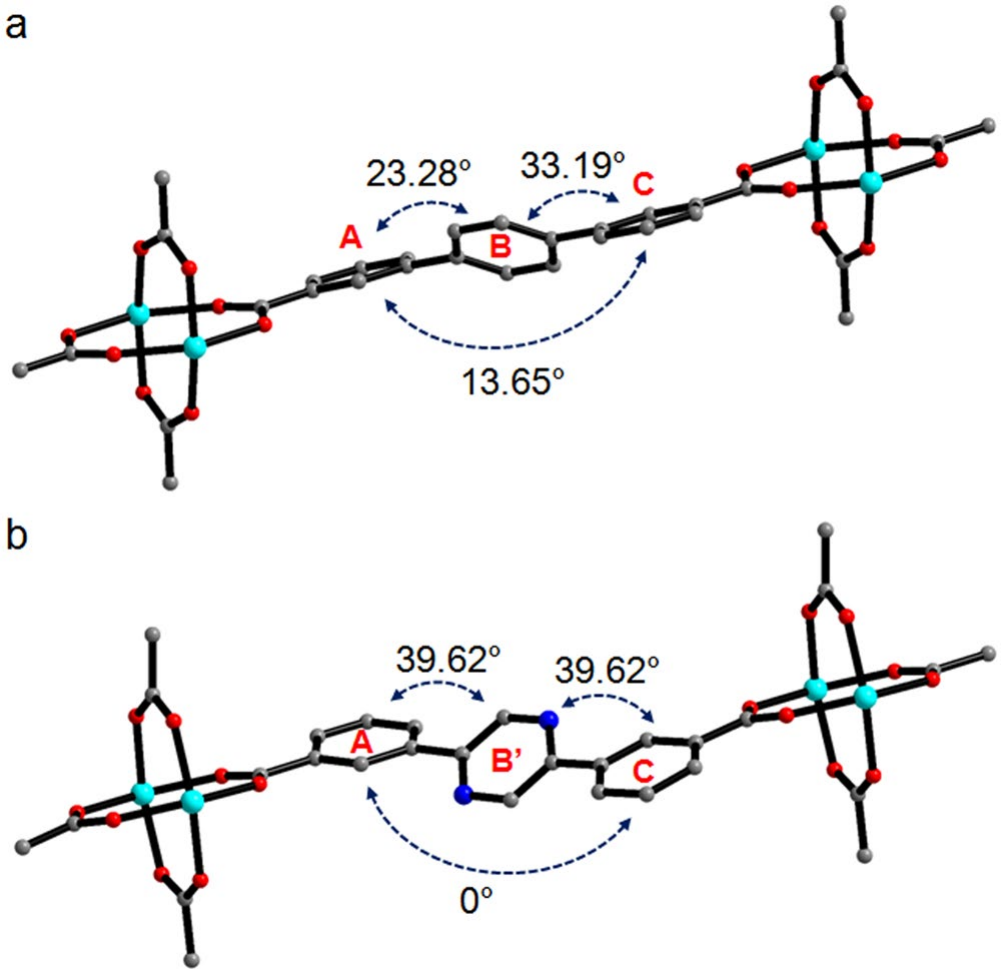
Comparison of the tilting angles of the ligands. (**a**) 3,3′-TPDC of Zn-MOF **1**. (b) 3,3′-PDBA of Zn-MOF **2**.

Interestingly, the 2D sheets are interlocked with two other neighboring 2D sheets to form an infinite 3D framework (Figs 3 and S3). It is worth mentioning that Zn-MOF **2** forms a 3D framework, while Zn-MOF **1** only forms 2D sheets without interlocking, despite their similar metal-ligand connectivity^13^. Subtle variation of the bridging ligand from 3,3′-TPDC to 3,3′-PDBA dramatically changes the framework structure. Both MOFs have similar frameworks containing paddle-wheel Zn_2_(CO_2_)_4_ SBUs, but the titling angles of the three rings of 3,3′-TPDC and 3,3′-PDBA are considerably different (Fig. 4). For 3,3′-TPDC, rings A and C are planar to each other, and ring B is tilted from both rings with a titling angle of 39.62°. For 3,3′-PDBA, the three rings are tilted away from each other with angles between A and B’, between B’ and C, and between A and C of 23.28°, 33.19°, and 10.65°, respectively. This indicates that 3,3′-PDBA containing a nitrogen-rich pyrazinyl ring is more tilted than 3,3′-TPDC, and this different titling behavior provides a different framework structure.

Both elemental analysis and thermogravimetric analysis (TGA) confirmed the stoichiometry of Zn-MOF **2**, as shown in Fig. S4. As-prepared Zn-MOF **2** showed a gradual weight loss until a temperature of 383 °C. This weight loss corresponds to the complete loss of two DMF solvates and a hydrate captured in the framework. The continuous increase of the temperature led to a more rapid weight loss, and this may correspond to decomposition of the bridging ligands and DABCO ligands. The purity of the bulk sample was confirmed by powder X-ray diffraction (PXRD). The PXRD pattern of as-prepared Zn-MOF **2** matches very well with the simulated pattern from X-ray crystallography, as depicted in Fig. S5.

**Figure 5:**
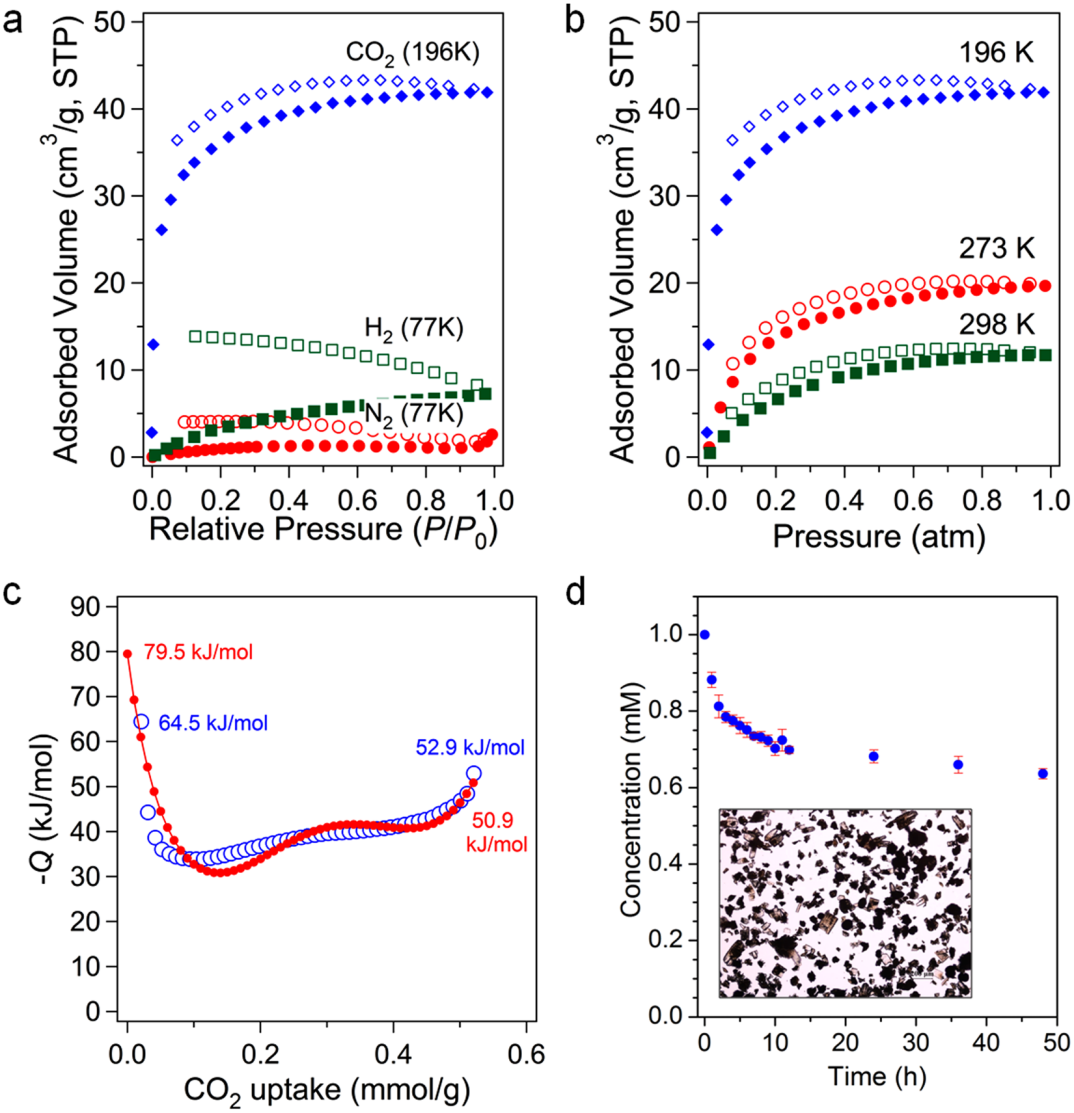
Gas sorption isotherms, isosteric heats of adsorption, and iodine encapsulation. (**a**) Adsorption/desorption isotherms of CO_2_, N_2_, and H_2_ for evacuated Zn-MOF **2**. (**b**) CO_2_ adsorption/desorption isotherms at three different temperatures. (**c**) Isosteric heats of CO_2_ adsorption calculated from the data at 273 and 298 K using the Clausius-Clapeyron equation (open blue symbols) and virial method (solid red symbols). (**d**) Timecourse of iodine encapsulation in cyclohexane. Measurements are performed in triplicate. The inset shows the digital photograph of **I**_**2**_ **@2**.

Zn-MOF **2** maintained its framework structure after drying at 393 K, as determined by the PXRD pattern (Fig. S5). Therefore, gas sorption analyses for N_2_, CO_2_, and H_2_ were performed by a standard volumetric method after drying at 393 K. N_2_ adsorption/desorption analysis at 77 K for evacuated Zn-MOF **2** did not show meaningful sorption of N_2_, as depicted in Fig. 5. H_2_ sorption also revealed that the uptake amount is negligible at 77 K (7.5 cm^3^ g^−1^). However, CO_2_ adsorption/desorption analysis exhibited 41.8 cm^3^ g^−1^ (1.87 mmol g^−1^) of uptake at 196 K. Thus, Zn-MOF **2** showed sorption selectivity for CO_2_ over N_2_ and H_2_. The hysteretic behaviors between adsorption and desorption isotherms for CO_2_ may come from the shape of 1D micropores. The micropores are not perfectly cylindrical geometry as evidenced from the Connolly surfaces shown in Fig. 3c and d. There are windows interconnecting cage-like pores. The aperture dimension of the window is slightly smaller than that of the cage-like pore. This pore geometry tends to induce the hysteretic sorption behaviors. The selective sorption of CO_2_ over other gases are important for the reduction of main greenhouse gas^1,2,15,16^. The pore size of Zn-MOF **2** was estimated from the CO_2_ adsorption isotherm as shown in Fig. S6. The Zn-MOF **2** shows bimodal porosity. The micropore dimensions are 0.56 and 0.44 nm corresponding to the large pore ‘A’ and small pore ‘B’, respectively. Increasing the measurement temperature led to a gradual decrease of the sorption amount of CO_2_: 19.7 cm^3^ g^−1^ (0.88 mmol g^−1^) at 273 K; 11.7 cm^3^ g^−1^ (0.52 mmol g^−1^) at 298 K. The isosteric heats of CO_2_ adsorption were calculated by two different methods for accurate determination: the Clausius-Clapeyron equation^17^ and virial method^17,18^ as shown in Figs 5c and S7. Each method has its own drawbacks^17^. In the calculation using the virial-type equation for curve fitting of the adsorption isotherms, it has been stressed that the standard errors of the polynomial coefficients should be as small as possible for good estimation of the adsorption enthalpy^19^. The correct choices of suitable polynomial coefficients are also very important^17^. The low surface coverage heat of CO_2_ adsorption calculated from the adsorption data at 273 and 298 K by the Clausius-Clapeyron equation is a high value of 64.5 kJ mol^−1^. The zero surface coverage value estimated using the virial method indicated an even higher value (79.5 kJ mol^−1^). In both cases, the heats of adsorption rapidly dropped once the uptake amount increased. However, further increase of the uptake amount gradually increased the heats of adsorption. This behavior can be attributed to the fact that a preadsorbed adsorbate induces a stronger interaction with the newly entering adsorbate. We are unable to propose the interaction between CO_2_ and DABCO site is exactly 1:1 because we do not have calculation results. Furthermore, there are also competing pyrazinyl Lewis basic sites from 3,3′-PDBA ligands to interact with CO_2_ molecules. Therefore, the rigorous theoretical investigation may give the information of the precise adsorption sites. Overall, these results clearly suggest that the high zero surface coverage heat of adsorption can be attributed to the nitrogen-rich environment of the 1D channels. The 3° amine-functionalized MOFs usually show high zero surface coverage heat of CO_2_ adsorption in the range 77–96 kJ mol^−1^ ^20^.

**Figure 6:**
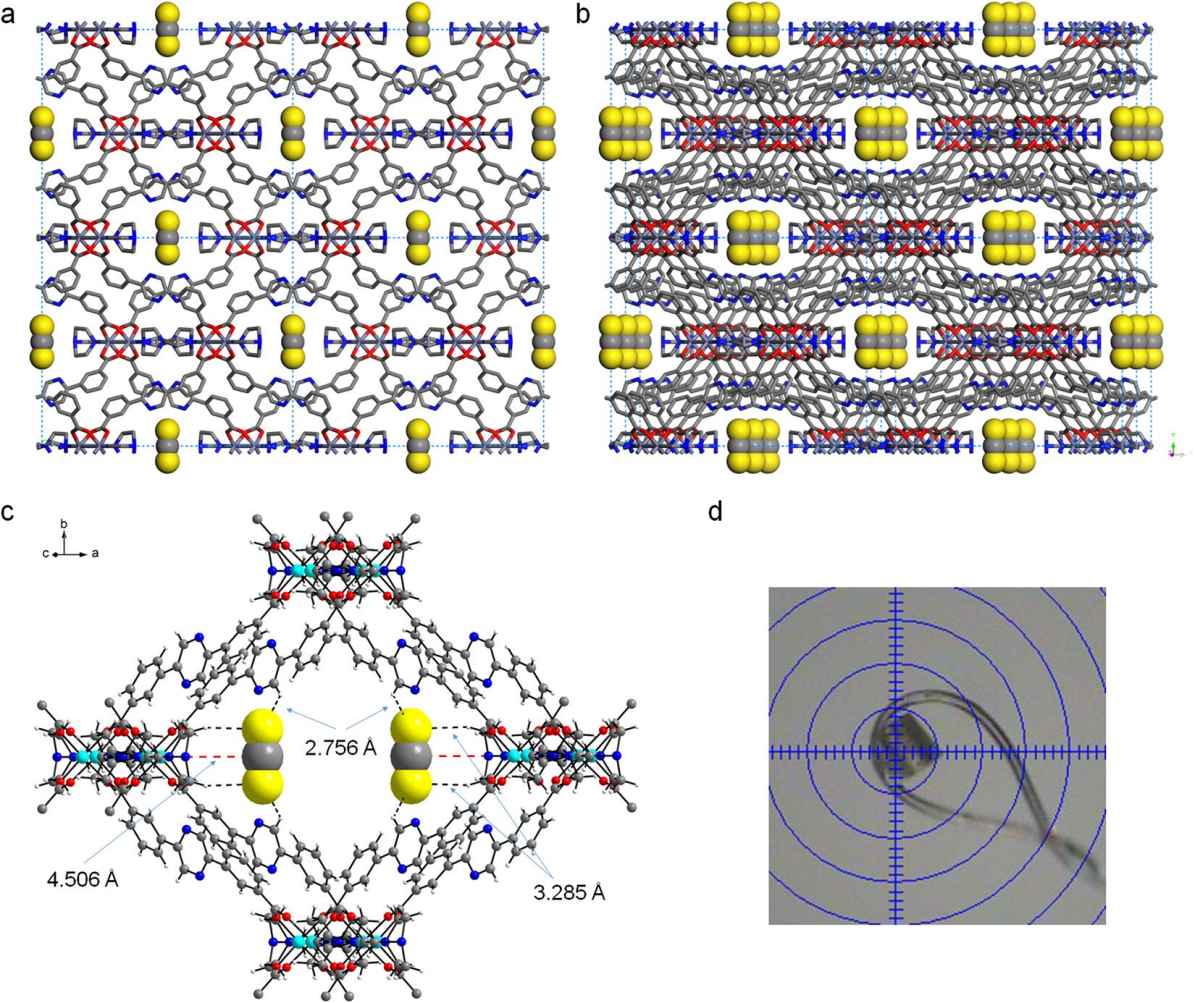
CS_2_ capture and crystal structure. (**a**) Structure of **CS**_**2**_ **@2** along the *c*-axis. (**b**) The corresponding tilted view. Hydrogen atoms are removed for clarity. CS_2_ molecules are shown in a CPK model. (**c**) Key intermolecular contact distances between the framework and CS_2_ molecule are shown. Only a single 2D layer is shown for clarity. (**d**) The yellow crystal of **CS**_**2**_ **@2** during the diffraction data collection.

The porous MOFs are often suited for effective encapsulation of potentially harmful volatile solids, liquids, and other guest molecules^21–28^. In this regard, we also investigated the encapsulation abilities of as-prepared Zn-MOF **2** using solid iodine and liquid CS_2_. Iodine is a volatile solid and potentially toxic^29^. CS_2_ is also a very toxic volatile liquid^30^. The time-dependent iodine capture was periodically monitored by analyzing the supernatant of reaction mixture in cyclohexane by UV/Vis spectroscopy, as shown in Fig. 5d. Based on these experiments, 0.20 mmol of iodine was encapsulated per mmol of Zn-MOF **2**. Colorless **2** changed into brown-colored crystals after iodine encapsulation. When the framework was exposed to liquid-phase CS_2_, the CS_2_ molecules were also effectively captured in the 1D micropores. Despite the same molecular geometry as CO_2_, the C = S bond in CS_2_ is much less polar compared with the C = O bond in CO_2_ due to the very small electronegativity difference between C (E.N. = 2.55) and S (E.N. = 2.58) atoms^31^. Furthermore, the C atom in CS_2_ is an electronegative center, unlike the electropositive C atom in CO_2_^32^. To probe the locations of the CS_2_ molecules in **CS**_**2**_ **@2**, the crystal structure of **CS**_**2**_ **@2** was obtained at 170 K, as depicted in Fig. 6. The occupancies of CS_2_ were fixed to obtain the best fit with the largest residual peaks (Table 1). The closest contacts between S (CS_2_)···H-C (3,3′-PDBA) and S (CS_2_)···H-C (DABCO) are 2.756 and 3.285 Å, respectively (Fig. 6c). The former contact distance is much smaller than the sum of the van der Waals radii of the two elements: S (1.80 Å) and H (1.20 Å)^33^. The latter contact distance is slightly longer than the sum of the van der Waals radii of the two elements. In contrast, the contact distance between C (CS_2_)···N (DABCO), 4.506 Å, is rather longer than these distances. Thus, the packing of nonpolar CS_2_ molecules in the 1D channels shown in Fig. 6 reveals that the CS_2_ molecules mainly interact with the framework through van der Waals contacts. It is interesting to note that the 1D channels can also effectively capture CS_2_ through van der Waals interactions despite the nitrogen-rich environment of the channels. The encapsulation amount of CS_2_ was calculated to be 8.2 wt% based on TG curve of **CS**_**2**_ **@2** as shown in Fig. S4.

**Table 1:** Crystallographic data for Zn-MOF **2** and **CS**_2_@**2**.

	Zn-MOF 2	CS_2_@2
Empirical formula	C_45_H_38_N_7_O_8_Zn_2_	(C_45_H_35_N_7_O_8_Zn_2_)·(CS_2_)_0.1_
Formula weight	935.56	940.16
Temperature (K)	296(2)	170(2)
Wavelength (Å)	0.71073	0.71073
Space group	C2/m	C2/m
a (Å)	26.1114(14)	26.0295(14)
b (Å)	21.1334(14)	21.2174(14)
c (Å)	8.5694(5)	8.4856(5)
α (°)	90.00	90.00
β (°)	101.379(5)	101.109(5)
γ (°)	90.00	90.00
Volume(Å^3^)	4635.9(5)	4598.6(5)
Z	4	4
Density (calc.) (Mg/m^3^)	1.340	1.347
Absorption coeff. (mm^−1^)	1.092	1.101
Crystal size (mm^3^)	0.20 × 0.10 × 0.04	0.160 × 0.120 × 0.080
Reflections collected	37041	63404
Independent reflections	4077 [R(int) = 0.2444]	5714 [R(int) = 0.2363]
Data/restraints/parameters	4077/6/281	5714/13/285
Goodness-of-fit on F^2^	1.043	0.965
Final R indices [I > 2σ(I)]	R_1_ = 0.1059, wR_2_ = 0.2266	R_1_ = 0.1014, wR_2_ = 0.2382
R indices (all data)	R_1_ = 0.1959, wR_2_ = 0.2648	R_1_ = 0.2140, wR_2_ = 0.2946
Largest diff. peak and hole (e.Å^−3^)	1.076 and −0.624	1.885 and −0.862
CCDC number	1491781	1491783

**Figure 7:**
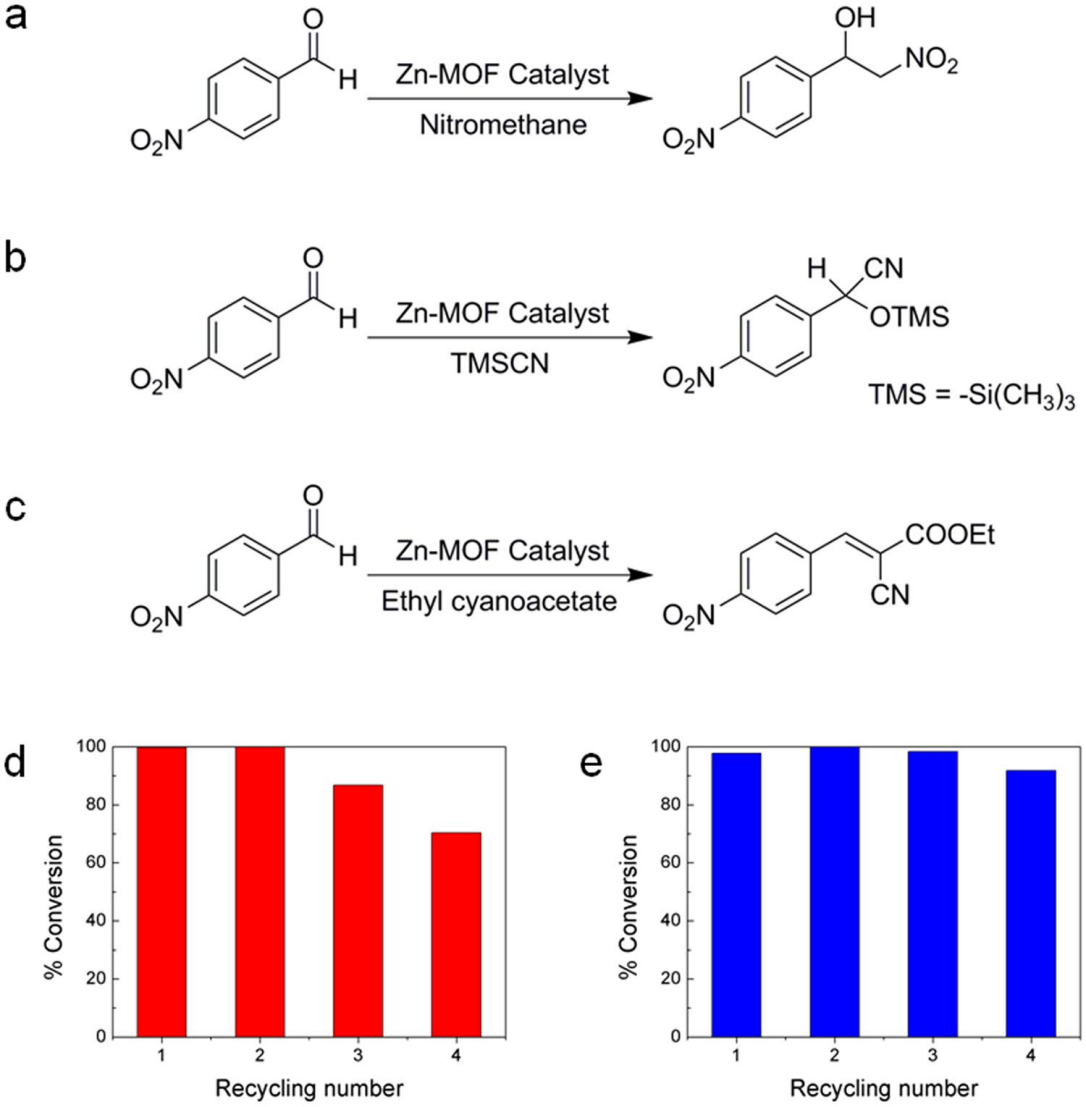
Heterogeneous catalysis by Zn-MOF 2. (**a**) Nitroaldol reaction, (**b**) cyanosilylation, and (**c**) Knoevenagel condensation reaction. Recycling experiments for cyanosilylation (**d**) and Knoevenagel condensation (**e**).

Heterogeneous catalytic systems based on MOFs have several advantages over other systems. MOFs with well-defined porosity and functionality can be employed to target catalysis of a substrate with a specific size and/or shape^7,14,34–36^. The active sites located inside the MOF channels closely resemble the active sites found in enzymes. The large surface area is also beneficial for efficient catalysis. Furthermore, the robust frameworks are often suitable as highly recyclable catalytic systems with easy and efficient recovery of catalysts through simple filtration or centrifugation^14^. The 3° amine-based Lewis basic sites of DABCO in the 1D channels of Zn-MOF **2** are ideal active catalytic sites. To investigate the catalytic activities of the dangling DABCO ligands, three different Lewis-base-catalyzed model reactions such as nitroaldol (Henry) reaction, cyanosilylation, and Knoevenagel condensation were tested, as shown in Fig. 7. These aldol-type C–C bond forming reactions are frequently employed as model reactions for Lewis base catalysts^37–39^. The respective conversions after certain periods of reaction time for the nitroaldol reaction, cyanosilylation, and Knoevenagel condensation of 4-nitrobenzaldehyde are 52%, 93%, and 100% under the same ratios of catalyst to substrate. These results confirm that the 3° amine sites of the dangling DABCO ligands are active heterogeneous Lewis base catalysis. Since the DABCO sites are 3° amine-based Lewis basic sites, they are good at deprotonating nitromethane or ethyl cyanoacetate to generate reactive nucleophiles in nitroaldol reaction or Knoevenagel condensation. In the case of cyanosilylation, the DABCO sites are thought to activate TMSCN through the formation of hypervalent pentacoordinate silicate species in which the nucleophilicity of cyano group is enhanced^40^. Both cyanosilylation and Knoevenagel condensation reactions exhibited relatively higher reaction conversions than the nitroaldol reaction. In the cyanosilylation, the resulting cyanohydrin trimethylsilyl ethers are important industrial intermediates for various value-added products^41,42^. The Knoevenagel condensation reaction also produces valuable fine chemicals and pharmaceutical products^38^. Thus, we investigated the recyclabilities of the Zn-MOF **2** catalytic system for both reactions. After four recycles, the cyanosilylation reaction showed moderate deactivation of the catalyst, i.e., 70% relative to the first reaction. The pore blocking by the generated product with a very low solubility may be responsible for the gradual decrease of conversions^43^. In contrast, the catalyst showed almost constant activity in the Knoevenagel condensation (94% after the 4th recycle) if we consider a small amount of catalyst was continuously lost during recovery. The retrieved catalyst after four recycles maintained its original framework structure, as evidenced by the PXRD pattern (Fig. S5).

Other aromatic aldehyde substrates with different substituents were also tested for the Knoevenagel condensation as summarized in Table S1. Depending on the substituents, the reaction conversions varied due to different electronic and steric effects. For instance, the reactivity order of 4-nitrobenzaldehyde (100%), 4-hydroxybenzaldehyde (49%), 4-methylbenzaldehyde (26%), and 4-methoxybenzaldehyde (~0%) is similar to the previous results catalyzed by -NH_2_ group-containing [{Cd_2_(L-glu)_2_(bpe)_3_(H_2_O)}·2H_2_O] (L-glu = L-glutamate dianion, bpe = 1,2-bis(4-pyridyl)ethylene)^44^. Thus, the 4-nitrobenzaldehyde with a good electron withdrawing substitutent at the *para*-position displayed the highest reactivity. As Zn-MOF **2** possesses bimodal porosity with pore size dimensions of 0.56 and 0.44 nm, even the larger pore size (0.56 nm) may not be large enough for the catalytic substrates to reach the active sites (DABCO). However, we speculate that the interlocked frameworks of Zn-MOF **2** can adjust their pore sizes in solution through flexible motions. It is really interesting to see the substrate size-dependent catalytic activities for the Knoevenagel condensation. For example, the more sterically demanding 2-nitrobenzaldehyde and 3-nitrobenzaldehyde did not show any conversion (~0%). While the reaction of the least sterically demanding 4-nitrobenzaldehyde underwent completion (~100%) under the same reaction conditions. These results clearly suggest that the catalytic reactions mainly occurred inside the channels of Zn-MOF **2**.

## Conclusion

In this study, we demonstrated a rational strategy for the generation of a nitrogen-rich confined space in a MOF by utilizing a *C*_2h_-symmetric N-containing bridging ligand. A 3D Zn-MOF, [Zn_2_(3,3′-PDBA)_2_(DABCO)_1.5_]·2DMF·H_2_O, containing the new pyrazine-derived 3,3′-(pyrazine-2,5-diyl)dibenzoic acid (3,3′-PDBA) bridging ligand and DABCO was prepared and fully characterized by X-ray crystallography. The Zn-MOF formed an interesting 3D framework consisting of mutually interlocked 2D layers. The uncoordinated N atoms of both the dangling DABCO and 3,3′-PDBA ligands efficiently generated a Lewis basic nitrogen-rich environment in the 1D channels. Based on the gas sorption analysis, the Zn-MOF showed selective adsorption of CO_2_ over N_2_ and H_2_ at low pressure due to the basic N atom sites in the channels. The Lewis basic uncoordinated N atoms of both DABCO and 3,3′-PDBA may have synergetic effects in showing a very high zero surface coverage heat of CO_2_ adsorption (79.5 kJ mol^−1^). The Zn-MOF was demonstrated as a recyclable heterogeneous catalyst for cyanosilylation and Knoevenagel condensation, as the structural robustness of the Lewis base catalyst maintained its catalytic activity for several repeated reactions. Therefore, the Zn-MOF displayed good CO_2_ selectivity and Lewis base catalytic activity.

## Experimental Methods

### Preparation of 3,3′-PDBA

2,5-Dibromopyrazine (0.714 g, 3.0 mmol, Sigma-Aldrich), 3-(methoxycarbonyl)phenylboronic acid (1.152 g, 6.4 mmol, TCI), tetrakis(triphenylphosphine)palladium(0) (0.102 g, 0.088 mmol, Strem) were dissolved in a mixture of deoxygenated toluene and ethanol (140 mL, 100:40% v/v) and a 2.0 M sodium carbonate aqueous solution (60 mL) under a nitrogen atmosphere. The mixture was heated at 85 °C for 48 h with stirring. The reaction mixture changed color from yellow to brown during the reaction. After the reaction, the solvent was evaporated to dryness using a rotary evaporator. The solid residue was poured into a 5% aqueous sodium bicarbonate solution (150 mL) and extracted with chloroform three times (150 mL × 3). The organic phase was separated and the solvent was removed under vacuum. The crude solid was purified by silica gel column chromatography (gradient elution starting from hexane:chloroform = 5:1 v/v). Yield was 0.70 g. ^1^H NMR (400.13 MHz, CDCl_3_, *δ*): 9.16 (2 H, *s*), 8.75 (2 H, *t*, 1.6 Hz), 8.30 (2 H, *dt*, 1.7 Hz, 7.9 Hz), 8.17 (2 H, *dt*, 1.4 Hz, 7.7 Hz), 7.63 (2 H, *t*, 7.8 Hz), 4.45 (4 H, *q*, 7.1 Hz), 3.99 (6 H, *s*), 1.45 (6 H, *t*, 7.0 Hz) ppm. ^13^C NMR (100.60 MHz, CDCl_3_, *δ*): 166.7, 150.1, 141.3, 136.5, 131.1, 131.1, 130.9, 129.3, 127.9, 52.4 ppm. The free acid was obtained by acid hydrolysis. The purified methyl ester compound (0.50 g) was dissolved in ethanol (20 mL) and a 6.0 M sodium hydroxide solution (10 mL). The mixture was stirred for 48 h at room temperature. The mixture was neutralized with 12.0 M hydrochloric acid until pH 4.0, and then, the solid was filtered and washed with a copious amount of distilled water. The white solid was dried for 24 h at 80 °C and subsequently dried under high vacuum. Hydrolysis yield was 95%. ^1^H NMR (400.13 MHz, DMSO-*d*_6_, *δ*): 9.42 (2 H, *s*), 8.77 (2 H, *t*, 1.4 Hz), 8.46 (2 H, *dt*, 1.4 Hz, 8.0 Hz), 8.08 (2 H, *dt*, 1.3 Hz, 7.9 Hz), 7.71 (2 H, *t*, 7.6 Hz) ppm. ^13^C NMR (100.60 MHz, DMSO-*d*_6_, *δ*): 166.9, 149.1, 141.3, 135.9, 131.7, 130.7, 130.5, 129.4, 127.3 ppm.

### Preparation of [Zn_2_(3,3′-PDBA)_2_(DABCO)_1.5_]·2DMF·H_2_O (2)

Zn(NO_3_)_2_·6H_2_O (0.030 g, 0.1 mmol, Aldrich), 3,3′-PDBA (0.032 g, 0.1 mmol), and 1,4-diazabicyclo[2,2,2]octane (DABCO) (0.006 g, 0.05 mmol, Aldrich) were dissolved in 5 mL of DMF and heated at 120 °C for 4 d. Transparent colorless block crystals were retrieved by filtration and washed with DMF. The crystals were dried in air (~ 0.024 g). High-quality block crystals were directly chosen for single-crystal X-ray crystallography from the as-prepared sample. Analytical analysis calculated for C_51_H_54_N_9_O_11_Zn_2_ (1099.81): C 55.70, H 4.95, N 11.46; found C 55.27, H 4.40, N 10.67%.

### Encapsulation of CS_2_

As-prepared Zn-MOF **2** (10 mg) was immersed in CS_2_ (10 mL) and gently shaken for 7 d at room temperature. The resulting **CS**_**2**_ **@2** was retrieved by filtration and dried in air at room temperature.

### Encapsulation of I_2_

As-prepared Zn-MOF **2** (20 mg) was soaked in 10 mL of a 1.0 mM cyclohexane solution of iodine. Aliquots of the solution (0.5 mL) were periodically transferred to a microtube and spun at an angular speed of 13,000 rpm for 10 min. After centrifugation, the supernatant of the solution (0.2 mL) was taken and diluted with fresh cyclohexane (1.8 mL) for UV/Vis spectroscopic analysis.

### Catalytic nitroaldol (Henry) reaction

Reagent-grade nitromethane was used without further purification. The reaction mixture of Zn-MOF **2** (17.3 mg, 0.0157 mmol), 4-nitrobenzaldehyde (4-NBA, 0.1511 g, 1.0 mmol), and nitromethane (10 mL) in a screw-capped vial was heated under constant stirring (200 rpm) at 60 °C for 120 h. The reaction mixture was filtered through a glass frit, and the solid residue was washed with chloroform and acetone. The solvent was removed from the filtrate by rotary evaporation, and the residue was dried under high vacuum and then completely dissolved in CDCl_3_ (10 mL). The product was analyzed by ^1^H NMR spectroscopy. Distinct chemical shifts were observed for the aromatic hydrogen atoms of the product. The signals were assigned by comparing the chemical shifts observed for the authentic sample.

### Catalytic cyanosilylation reaction

A reaction mixture of Zn-MOF **2** (17.3 mg, 0.0157 mmol), 4-NBA (0.1511 g, 1.0 mmol), and trimethylsilyl cyanide (TMSCN, 0.125 mL, 1.0 mmol) in dry toluene (10 mL) in a screw-capped vial was heated under constant stirring (200 rpm) at 50 °C for 24 h. The reaction mixture was then filtered through a glass frit, and the solid was washed with chloroform and acetone. The solvent was removed from the filtrate by rotary evaporation, and the residue was dried under high vacuum and then completely dissolved in a mixture of CDCl_3_ and acetone-*d*_6_ (~45 mL). An internal standard, 1,1,2,2-tetrachloroethane (1.0 mmol, Sigma-Aldrich), was added to the deuterated solvent. The product was analyzed by ^1^H NMR spectroscopy. A distinct chemical shift at 5.6 ppm was observed for the cyanohydrin trimethylsilyl ether product. The signals were assigned by comparing the chemical shifts observed for the product with literature values.

### Recycling test for cyanosilylation

The recycling test for the cyanosilylation of 4-NBA with TMSCN catalyzed by Zn-MOF **2** was performed with a 4-fold increased scale for easy recovery of the catalyst. Zn-MOF **2** (69.3 mg, 0.0630 mmol), 4-NBA (0.605 g, 4.0 mmol), TMSCN (0.50 mL, 4.0 mmol) in dry toluene (10 mL) in a screw-capped vial were heated under constant stirring (200 rpm) at 50 °C for 24 h. The work-up procedure was the same as the previous case. The air-dried recycled Zn-MOF **2** was employed for the next run of the reaction with new substrates under the same reaction conditions. A total of four reactions were repeated.

### Catalytic Knoevenagel condensation

A reaction mixture of Zn-MOF **2** (17.3 mg, 0.0157 mmol), an aldehyde of choice (1.0 mmol), and ethyl cyanoacetate (0.107 mL, 1.0 mmol) in dry toluene (10 mL) in a screw-capped vial was heated under constant stirring (200 rpm) at 50 °C for 48 h. The reaction mixture was filtered through a glass frit, and the solid residue was washed with chloroform and acetone. The solvent was removed from the filtrate by rotary evaporation, and the residue was dried under high vacuum and then completely dissolved in a mixture of CDCl_3_ and acetone-*d*_6_ (~10 mL). An internal standard, 1,1,2,2-tetrachloroethane (1.0 mmol, Sigma-Aldrich), was added to the deuterated solvent. The product was analyzed by ^1^H NMR spectroscopy by comparing the vinylic proton resonance signal approximately at 8.20 ppm and the signal of the internal standard at 5.95 ppm.

### Recycling test for Knoevenagel condensation

The recycling test for the Knoevenagel condensation reaction of 4-NBA with ethyl cyanoacetate catalyzed by Zn-MOF **2** was performed with a 4-fold increased scale for easy recovery of the catalyst. Zn-MOF **2** (69.3 mg, 0.0630 mmol), 4-NBA (0.605 g, 4.0 mmol), ethyl cyanoacetate (0.427 mL, 4.0 mmol) in dry toluene (10 mL) in a screw-capped vial were heated under constant stirring (200 rpm) at 50 °C for 48 h. The work-up procedure was the same as the previous case. The air-dried recycled Zn-MOF **2** was employed for the next run of the reaction with new substrates under the same reaction conditions. A total of four reactions were repeated.

### X-ray crystallography

The X-ray diffraction data for Zn-MOF **2** and **CS**_**2**_ **@2** were collected on a Bruker APEX-II diffractometer equipped with a monochromator and a Mo Kα (λ = 0.71073 Å) incident beam. A crystal of **2** was mounted on a glass fiber, and data were collected at room temperature. A crystal of **CS**_**2**_ **@2** was mounted on a silicon loop, and data were collected at 170 K. The CCD data were integrated and scaled using the Bruker-SAINT software package, and the structure was solved and refined using SHELXTL V6.12^45^. All hydrogen atoms were placed in the calculated positions. The crystallographic data for the two compounds are listed in Table 1. The selected bond distances and angles of the compounds are listed in Table 2. Structural information was deposited at the Cambridge Crystallographic Data Center (CCDC reference numbers are 1491781 for Zn-MOF **2** and 1491783 for **CS**_**2**_ **@2**).

**Table 2:** Selected bond distances for Zn-MOF **2** and **CS**_2_@**2**..

	Zn-MOF 2	CS_2_@2
Zn-O_CO2_ (Å)	2.018(7)–2.044(8)	2.043(4)–2.053(4)
Zn-N_DABCO_ (Å)	2.043(10), 2.053(13)	2.047(7), 2.048(8)

### Gas sorption analysis

Cryogenic N_2_ adsorption/desorption analysis was performed using a Belsorp-miniII instrument at 77 K (BEL Japan). The CHCl_3_-exchanged sample was obtained by shaking a CHCl_3_ suspension of the fresh as-prepared Zn-MOF **2** for 3 d. The CHCl_3_-exchanged sample was dried at 393 K under high vacuum for 2 h before the measurements. Low-pressure CO_2_ adsorption/desorption measurements were also performed using the same equipment at 196 K (2-propanol/dry ice bath), 273 K (ice bath), and 298 K (water bath). Ultrapure grade (99.999%) CO_2_ gas was used for the gas sorption experiments. A moisture trap was placed at the outlet of the CO_2_ gas cylinder to avoid moisture contamination during the measurements. The isosteric heats of CO_2_ adsorption were estimated using the subroutine implemented in the BEL Master software of the Belsorp-miniII equipment. The subroutine uses the Clausius-Clapeyron equation to estimate the adsorption enthalpies from the adsorption data measured at 273 and 298 K. Low-pressure H_2_ adsorption measurements were performed at 77 K on a Belsorp-miniII instrument. The equipment was calibrated using Cu-BTC (HKUST-1) as a reference material^12^. The as-made HKUST-1 activated at 393 K under high vacuum for 2 h showed a hydrogen uptake value of 2.23 wt% at 77 K and 1 bar. This value agrees well with the reported value (2.27 wt%) under the same measurement conditions^46^.

### Instrumentation

NMR spectra were recorded on a Bruker Ascend 400 (400.13 MHz for ^1^H and 100.60 MHz for ^13^C) spectrometer. ^1^H chemical shifts were referenced to the proton resonance signal resulting from the protic residue in the deuterated solvent. ^13^C chemical shifts were referenced to the carbon resonance signal resulting from the deuterated solvent. Thermogravimetric analysis was carried out on a TGA Q5000 instrument (TA Instruments) under a nitrogen atmosphere. Elemental analysis was performed at the Organic Chemistry Research Center, Sogang University (Seoul, Korea) using an EA1112 instrument (CE Instruments, Italy). Powder XRD spectra were obtained with a Bruker D8 Focus diffractometer (40 kV, 30 mA, step size = 0.02°). Optical microscopic images were collected on a Nikon Eclipse LV100POL microscope equipped with a DS-Fi1 CCD camera.

## Electronic supplementary material

Supporting Information
