# Supplementary material for: Selective carbon dioxide sorption and heterogeneous catalysis by a new 3D Zn-MOF with nitrogen-rich 1D channels
Source: Sci Rep. 2017 Dec 7;7:17185. doi: 10.1038/s41598-017-17584-8 (PMC5719397; doi:10.1038/s41598-017-17584-8)
Supplement: Supplementary file 1 — Supporting Information [file 41598_2017_17584_MOESM1_ESM.pdf]

Supporting Information

Selective carbon dioxide sorption and heterogeneous catalysis by a new 3D Zn-MOF with nitrogen-rich 1D channels

Hyun-Chul Kim,[1] Seong Huh,[*,1] Sung-Jin Kim,[2] and Youngmee Kim[*,2]

[1]*Department of Chemistry and Protein Research Center for Bio-Industry, Hankuk University of Foreign Studies, Yongin 17035, Korea*

[2]*Department of Chemistry and Nano Science, Ewha Womans University, Seoul 03760, Korea*

* To whom correspondence should be addressed. E-mail: shuh@hufs.ac.kr, ymeekim@ewha.ac.kr

Calculation of isosteric heats of CO_2 adsorption

(1) Method 1 (Clausius-Clapeyron equation):

The subroutine implemented in the BEL Master program (BEL Japan) was used. The adsorption data measured at 273 and 298 K were used.

(2) Method 2 (Virial method):

In order to calculate the isosteric heat of adsorption (Q_{st}) using virial-type of equation. The following Clausius-Clapeyron equation (1) was used for the estimation of Q_{st}.

$$-Q_{st} = -RT^2 \frac{\partial \ln(P)}{\partial T} \tag{1}$$

The following virial-type of equation (2) was used for the curve fitting of combined adsorption data obtained at 273 and 298 K (OriginPro 8.1).

$$\ln(P) = \ln(n) + \frac{1}{T}\sum_{i=0}^{j} a_i n^i + \sum_{i=0}^{k} b_i n^i \tag{2}$$

The resulting polynomial coefficients are used for the calculation of the following equation (3) to get adsorption enthalpy. R is the universal gas constant.

$$-Q_{st} = -R\sum_{i=0}^{j} a_i n^i \tag{3}$$

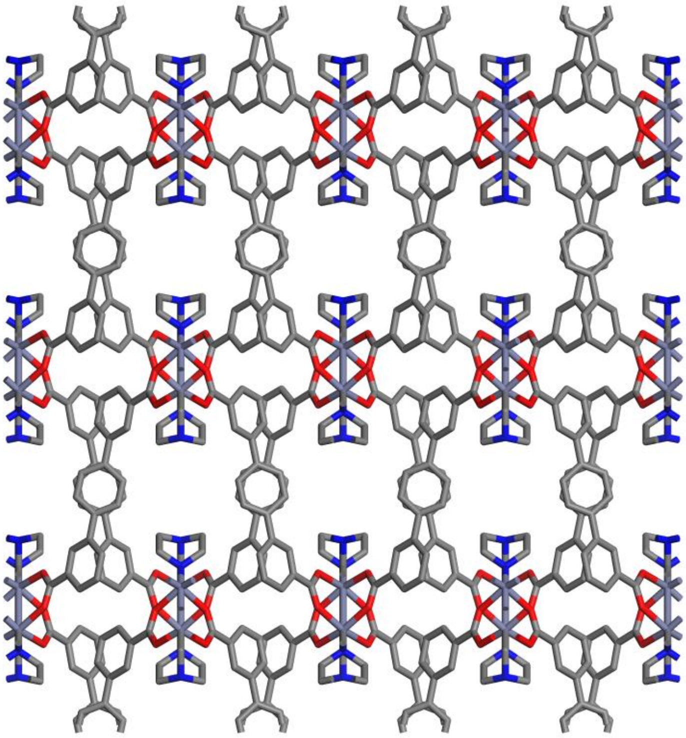

Figure S1. A view of the desolvated Zn-MOF **1** shown along the *a*-axis. Hydrogen atoms are omitted for clarity.

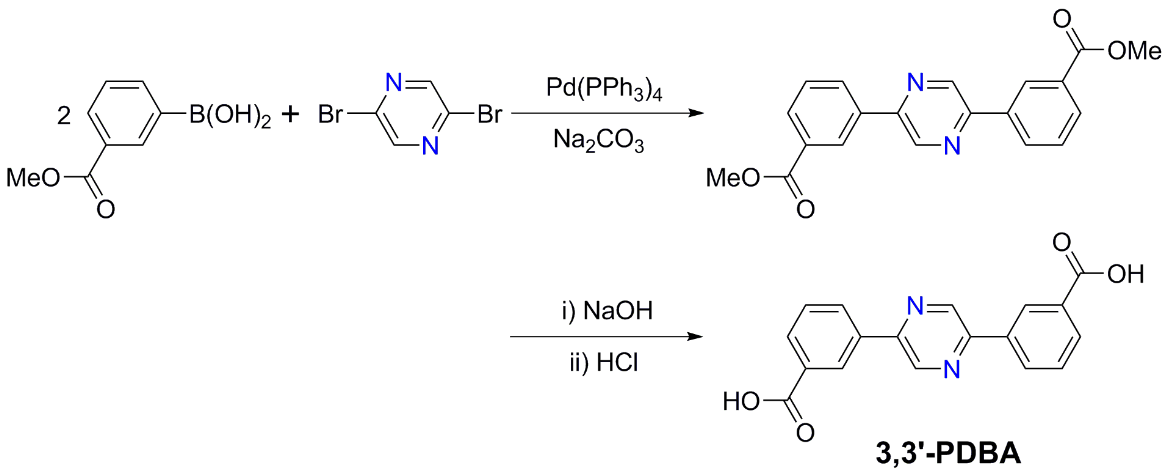

Figure S2. Preparation scheme for the new C_{2h}-symmetric 3,3'-PDBA bridging ligand by catalytic Suzuki coupling reaction.

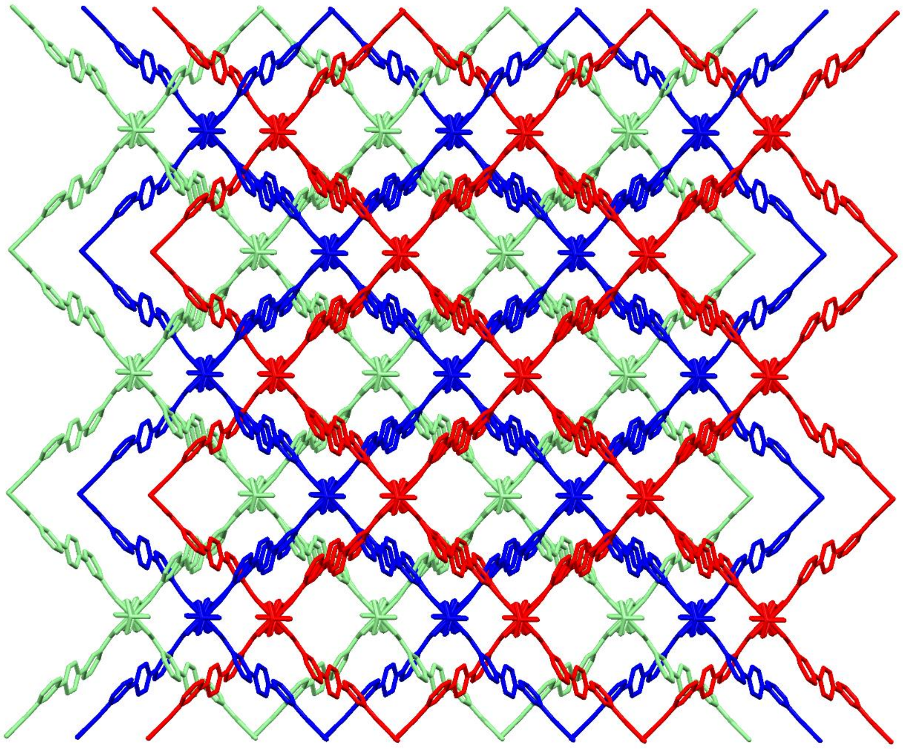

Figure S3. Interlocked structure of 3D Zn-MOF **2** shown along the *c*-axis. Different colours indicate different 2D sheets.

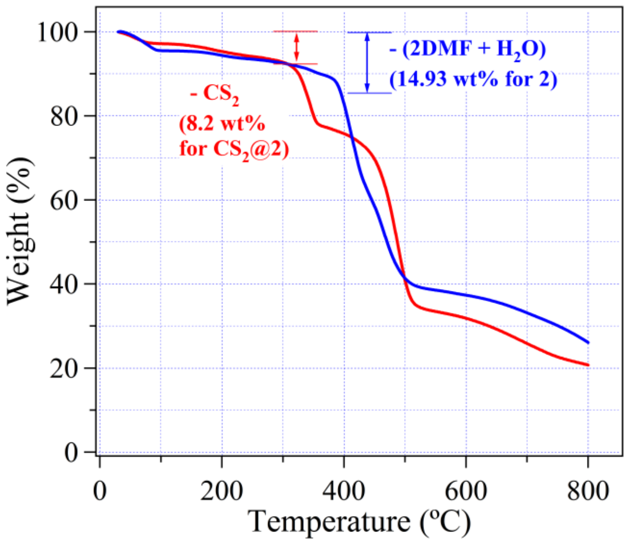

Figure S4. TG profiles for as-prepared Zn-MOF **2** (blue curve) and **CS_2@2** (red curve).

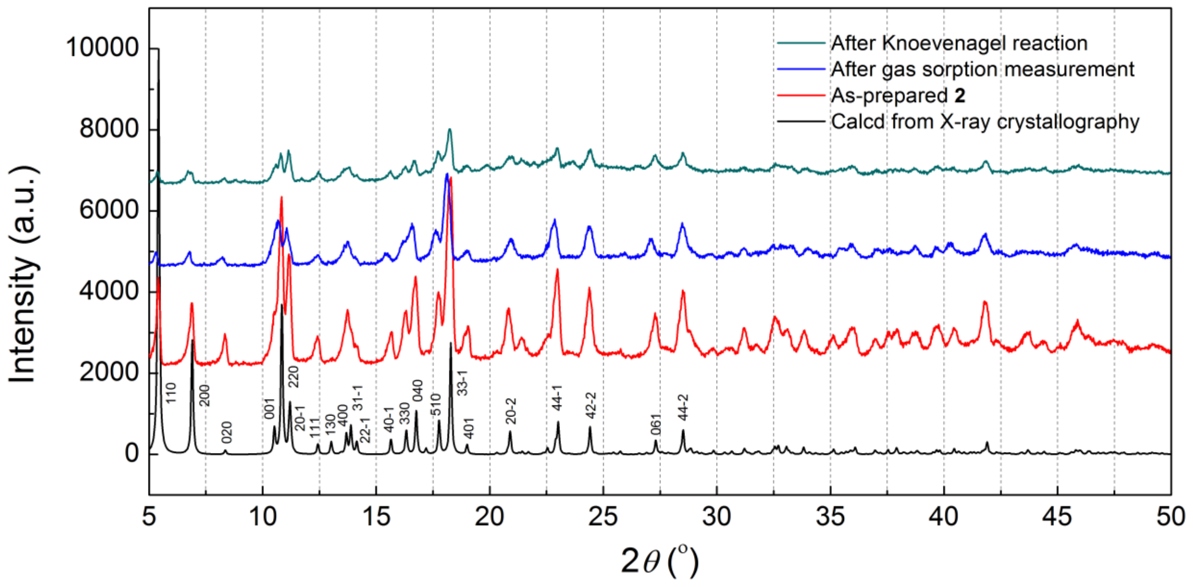

Figure S5. PXRD patterns of as-prepared Zn-MOF **2**, simulated pattern from X-ray crystallography, activated Zn-MOF **2**, and retrieved Zn-MOF **2** after four cycles of Knoevenagel condensation.

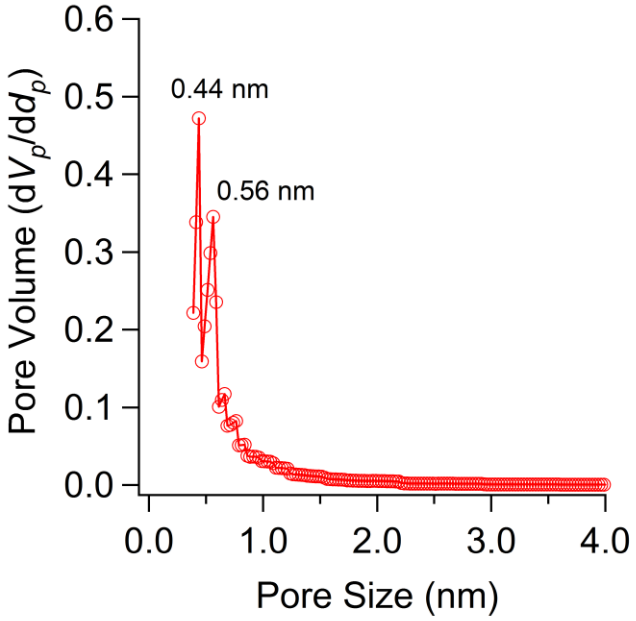

Figure S6. Pore size distribution curve for Zn-MOF **2** from CO_2 adsorption data at 196 K.

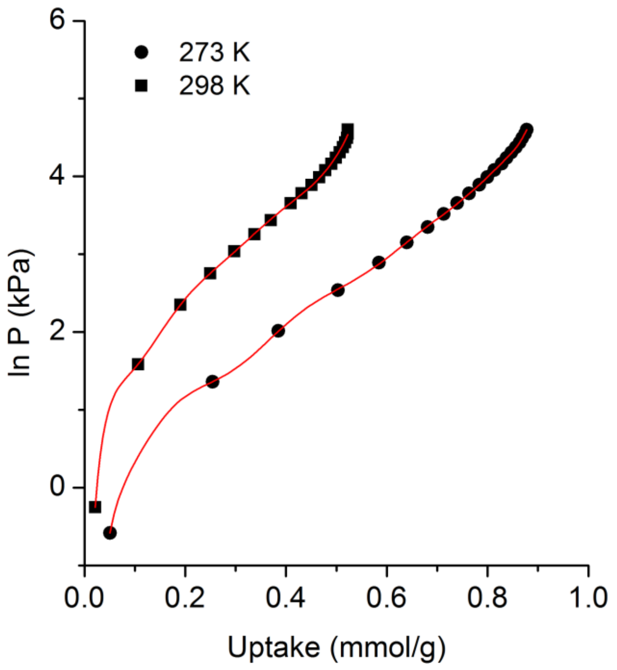

Equation	y = ln(x)+(1/T)*(A0+(A1*x)+(A2*x^2)+(A3*x^3)+(A4*x^4)+(A5*x^5)+(A6*x^6)+(A7*x^7)+(A8*x^8)+(A9*x^9)+(A10*x^10)+(A11*x^11))+(B0+(B1*x)+(B2*x^2)+(B3*x^3)+(B4*x^4))		
Adj. R-Square			0.99974
		Value	Standard Error
ln P	A0	-9562.18343	952.41316
ln P	A1	136242.99696	22401.12348
ln P	A2	-1.45622E6	226851.85025
ln P	A3	1.0912E7	1.88091E6
ln P	A4	-6.36101E7	1.10246E7
ln P	A5	2.6124E8	4.2227E7
ln P	A6	-7.17996E8	1.07928E8
ln P	A7	1.31122E9	1.86314E8
ln P	A8	-1.57559E9	2.15189E8
ln P	A9	1.19693E9	1.5961E8
ln P	A10	-5.21139E8	6.88082E7
ln P	A11	9.91135E7	1.31127E7
ln P	B0	34.11056	2.94064
ln P	B1	-336.48056	62.68522
ln P	B2	2130.57909	389.44325
ln P	B3	-5145.62247	907.81083
ln P	B4	4295.15101	714.01057
ln P	T	273	0
ln P	A0	-9562.18343	952.41316
ln P	A1	136242.99696	22401.12348
ln P	A2	-1.45622E6	226851.85025
ln P	A3	1.0912E7	1.88091E6
ln P	A4	-6.36101E7	1.10246E7
ln P	A5	2.6124E8	4.2227E7
ln P	A6	-7.17996E8	1.07928E8
ln P	A7	1.31122E9	1.86314E8
ln P	A8	-1.57559E9	2.15189E8
ln P	A9	1.19693E9	1.5961E8
ln P	A10	-5.21139E8	6.88082E7
ln P	A11	9.91135E7	1.31127E7
ln P	B0	34.11056	2.94064
ln P	B1	-336.48056	62.68522
ln P	B2	2130.57909	389.44325
ln P	B3	-5145.62247	907.81083
ln P	B4	4295.15101	714.01057
ln P	T	298	0

Figure S7. Virial fitting of the combined data for CO_2 adsorption at 273 and 298 K (OriginPro 8.1).

Table S1. Conversions of various aromatic aldehydes in the Knoevenagel condensation reaction with ethyl cyanoacetate catalyzed by Zn-MOF **2**.

Aldehydes	Conversions (%)
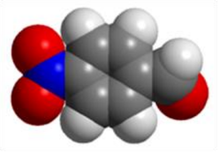 4-Nitrobenzaldehyde 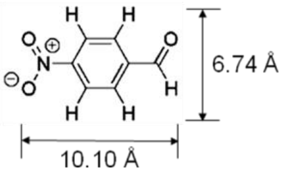	100
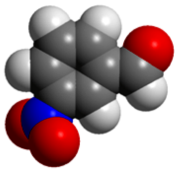 3-Nitrobenzaldehyde 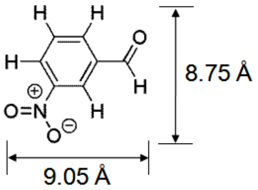	~0
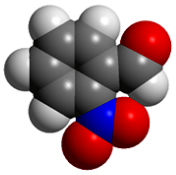 2-Nitrobenzaldehyde 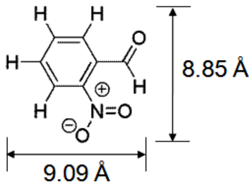	~0
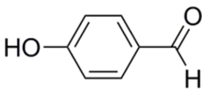 4-Hydroxybenzaldehyde	49
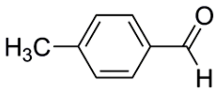 4-Methylbenzaldehyde	26
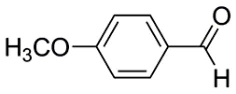 4-Methoxybenzaldehyde	~0
